# Pressure‐Induced Changes in Intracellular pH Facilitate the Myogenic Response of Rat Small Tail Arteries

**DOI:** 10.1111/apha.70309

**Published:** 2026-09-16

**Authors:** Ulrike Krien, Rudolf Schubert

**Affiliations:** ^1^ Physiology, Institute of Theoretical Medicine, Faculty of Medicine University of Augsburg Augsburg Germany

**Keywords:** arteries, intracellular pH, myogenic response, vasoconstriction

## Abstract

**Aim:**

The myogenic response contributes considerably to blood flow autoregulation. Intracellular pH (pH_i_) has a substantial impact on small artery contractility. However, the role of putative pH_i_ changes during myogenic constriction is unknown. Therefore, the hypothesis was tested that changes in pH_i_ are associated with and contribute to the myogenic response in small arteries.

**Methods:**

Rat small tail arteries were studied using isobaric myography and BCECF‐ as well as FURA‐2‐fluorimetry.

**Results:**

We found that in response to a pressure increase, pH_i_ decreased. It initially reached a peak and then recovered partly. The initial pH_i_ peak is augmented in bicarbonate‐free solution, but was not affected by inhibitors of Na^+^/H^+^ exchange. The pH_i_ recovery was not altered in bicarbonate‐free solution, but was reduced by inhibitors of Na^+^/H^+^ exchange. Proton fluxes were larger at 80 mmHg than at 10 mmHg. Bicarbonate‐free solution had no effect on proton fluxes at either 80 mmHg or 10 mmHg. Inhibitors of Na^+^/H^+^ exchange reduced proton fluxes only at 80 mmHg. Pressure‐induced changes in intracellular calcium were not affected by either bicarbonate‐free solution or inhibitors of Na^+^/H^+^ exchange.

**Conclusions:**

This study showed that in rat small tail arteries a pressure increase is accompanied by a decrease in pH_i_ that appears to facilitate the myogenic response. The results of this study suggest that the pressure‐induced immediate fall in pH_i_ is caused by a release of protons from the contractile machinery, and that the subsequent pH_i_ recovery is due to activation of Na^+^/H^+^ exchange.

## Practitioner Points

- The myogenic response, that is, the change in the diameter of small arteries and arterioles in response to a change in blood pressure, is one of the most important mechanisms by which blood perfusion of a given organ can be adapted to its metabolic demand, and plays an important role in health and disease.
- This study showed that a pressure increase is accompanied by a decrease in intracellular pH (pH_i_), which appears to facilitate the myogenic response. The data further suggest that the pressure‐induced immediate fall in pH_i_ is caused by a release of protons from the contractile machinery, and that the subsequent pH_i_ recovery is due to activation of Na^+^/H^+^ exchange.
- Changes in pH_i_ are not only associated with contractile responses of small arteries but are also related to changes in extracellular pH (acidosis, alkalosis). Therefore, changes in pH_i_ caused by acidosis and alkalosis may influence the changes in pH_i_ associated with the myogenic response. This interaction warrants further investigation in the future.

## 1 Introduction

In many organs, even large changes in perfusion pressure result in only small or negligible alterations in blood flow and capillary hydrostatic pressure. This autoregulation is one of the most important mechanisms that allows the blood perfusion of a given organ to be matched with its metabolic demand, despite variations in blood pressure. It has been suggested that myogenic control, that is, the myogenic response of small arteries and arterioles, contributes considerably to the regulation of blood flow, capillary hydrostatic pressure, and wall tension in response to changes in perfusion pressure in many vascular beds. In addition, alterations of the myogenic response are associated with several widespread diseases such as hypertension and diabetes (for reviews see [1, 2, 3, 4]). Due to its functional importance for health and disease, the study of the myogenic response is one of the most intensively researched topics in circulatory physiology.

In recent years, considerable effort has been devoted to the investigation of the mechanisms of the myogenic response. Briefly, it is now established that a pressure increase leads to membrane depolarization of smooth muscle cells, most likely due to an opening of stretch‐activated cation channels. The depolarization activates voltage‐operated calcium channels, causing an enhanced influx of extracellular calcium. Subsequently, activation of MLCK by calcium augments LC_20_ phosphorylation, leading to vessel constriction. Furthermore, a number of other signaling molecules and mechanisms seem to be involved, particularly mechanisms regulating the calcium sensitivity of the contractile apparatus, but these are still under discussion because of divergent opinions or incomplete evidence (for reviews see [1, 2, 3, 4, 5, 6, 7, 8]). The search for unknown mechanisms contributing to the myogenic response continues.

Besides calcium, another intracellular ion, H^+^, has attracted considerable attention in studies focusing on contractile mechanisms of small arteries. The intracellular pH (pH_i_) of vascular smooth muscle cells is determined by metabolic production and consumption of acid–base equivalents. Further, it is tightly controlled by pH‐buffers, mainly proteins and bicarbonate—also in conjunction with carbonic anhydrase—and by sarcolemmal pH_i_‐regulating ion exchangers, such as the Na^+^/${\mathrm{HCO}}_{3}^{-}$ co‐transport (mainly NBCn1), the Na^+^/H^+^‐exchange (mainly NHE1) and the Cl^−^/${\mathrm{HCO}}_{3}^{-}$‐exchange (see [9, 10, 11] and reviews [12, 13, 14, 15]). Nevertheless, changes in smooth muscle cell pH_i_ can occur in small arteries. For example, agonist‐induced contractions are accompanied by a decrease in pH_i_ in mouse [16, 17] and rat [18, 19, 20] mesenteric arteries. Due to the pH_i_‐controlling mechanisms mentioned above, pH_i_ changes are usually transient. After the initial pH_i_ perturbation, a recovery of pH_i_ takes place. Yet, these changes in pH_i_ have important functional consequences. The immediate response to a selective fall in pH_i_, irrespective of its cause, is an increase in tension in most preparations (for overview see [12, 13, 15, 21, 22]). In addition, tension is undergoing further changes during the pH_i_ recovery phase. Thus, pH_i_ has substantial impact on small artery contractility. Although the major mechanisms that generally determine the impact of pH_i_ on vessel contractility were largely established several years ago, the role of putative pH_i_ changes during myogenic constriction remains unknown to this date. Therefore, the hypothesis was tested that changes in pH_i_ are associated with and contribute to the myogenic response in small arteries.

## 2 Results

Rat tail small arteries exposed to a constant transmural pressure of 80 mmHg developed a spontaneous myogenic tone; vessel diameter decreased to 80.3% ± 1.7% (*n* = 30) of the fully relaxed state, where the latter was determined in calcium free solution at 80 mmHg.

### 2.1 Effect of Pressure Changes on Vessel Diameter and pH_i_

**FIGURE 1:**
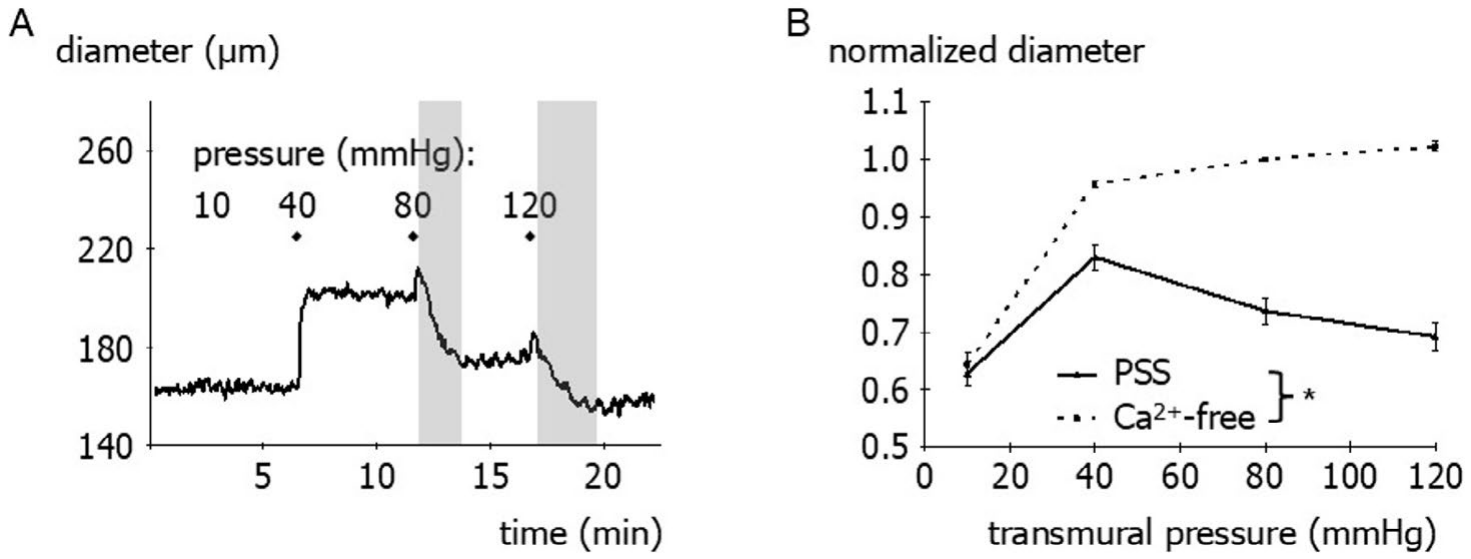
Effect of transmural pressure on vessel diameter. (A) Original recording of pressure‐induced diameter reaction in physiological saline solution (PSS). The gray area marks the period of active vessel constriction. (B) Summarized data of the effect of transmural pressure on vessel diameter in PSS and in calcium‐free solution. Steady state values of vessel diameter are shown at the respective pressure, normalized to the diameter of the fully relaxed vessel in calcium‐free solution at 80 mmHg. Repeated measures two‐way ANOVA (PSS vs. Ca^2+^‐free): *n* = 8 (PSS), *n* = 6 (Ca^2+^‐free); *p < 0.05.

In the physiological range of transmural pressure, vessels showed a typical myogenic response, that is, a sudden increase in pressure leads to a passive dilation of the vessel followed by an active constriction (Figure 1A) resulting in considerably smaller vessel diameters compared to calcium‐free conditions (Figure 1B).

**FIGURE 2:**
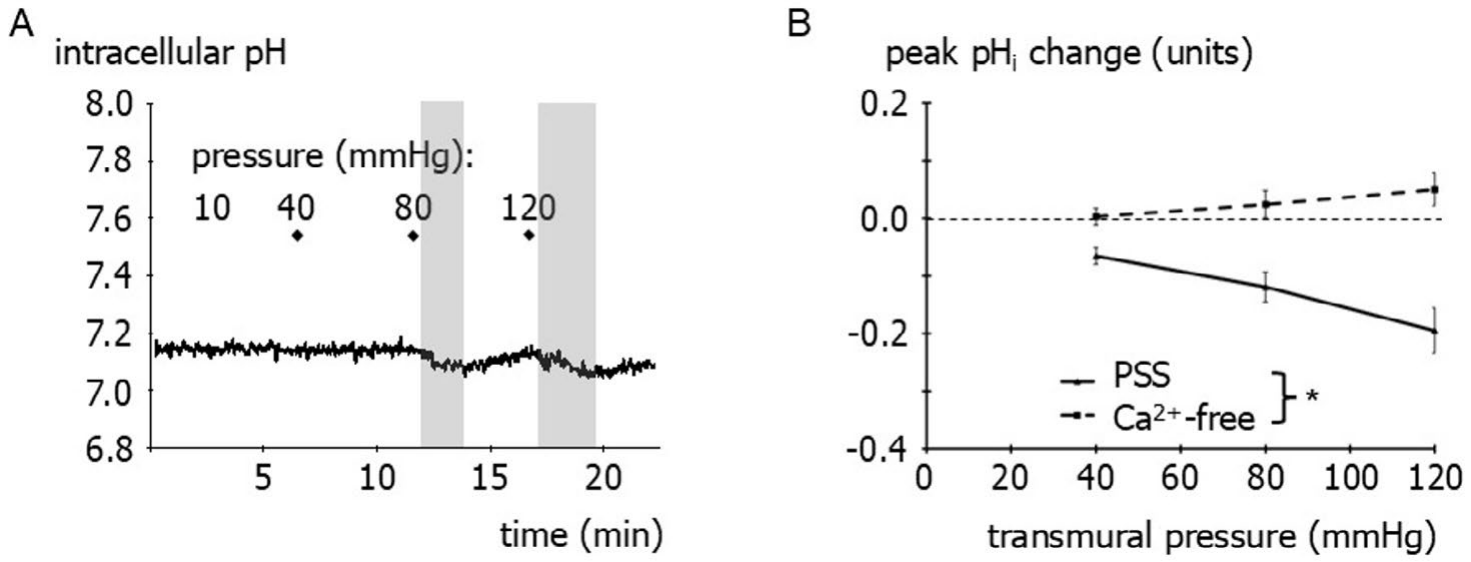
Effect of transmural pressure on intracellular pH. (A) Original record of pressure‐induced pH_i_ reactions in PSS. The gray area marks the period of active vessel constriction as shown in Figure 1A. (B) Summarized data of the effect of transmural pressure on peak pH_i_ change (determined relative to the pH_i_ at 10 mmHg) in PSS (solid line) and calcium free solution (dashed line). Repeated measures two‐way ANOVA (PSS vs. Ca^2+^‐free): *n* = 6; *p < 0.05.

The pressure‐induced diameter changes were accompanied by alterations of pH_i_. In response to a pressure‐increase, pH_i_ decreased continuously as long as the vessel constriction proceeded (Figures 1A and 2A, periods of active vessel constriction are marked by gray areas), with a similar time course as the accompanying vessel constriction. After reaching a peak, pH_i_ recovered partly (Figure 2A). At 10 mmHg, we did not detect a difference between pH_i_ in physiological saline solution (7.02 ± 0.07, *n* = 6) and in calcium‐free solution (7.03 ± 0.08, *n* = 6; *p* = 0.70; Mann–Whitney test). In the physiological range of transmural pressure, pH_i_ decreased with increasing pressure in physiological saline solution (*n* = 6; *p* < 0.05; one‐way ANOVA). In contrast, we did not detect any change in pH_i_ during pressure alterations in calcium‐free solution (*n* = 6; *p* = 0.13; one‐way ANOVA) (Figure 2B). Pressure‐induced changes in peak pH_i_ in physiological saline solution were considerably different from those under calcium‐free conditions (Figure 2B).

### 2.2 Effect of Inhibition of Sarcolemmal pH_i_ Regulating Mechanisms on Pressure‐Induced Changes of pH_i_

In order to gain insight into the mechanisms contributing to the pressure‐induced changes in pH_i_, sarcolemmal pH_i_ regulating mechanisms were inhibited. Under control conditions, the peak pH_i_ change was −0.10 ± 0.01 units (*n* = 24) upon a pressure increase from 10 to 80 mmHg. Then, pH_i_ recovered by 50.4% ± 4.0% (*n* = 24).

**FIGURE 3:**
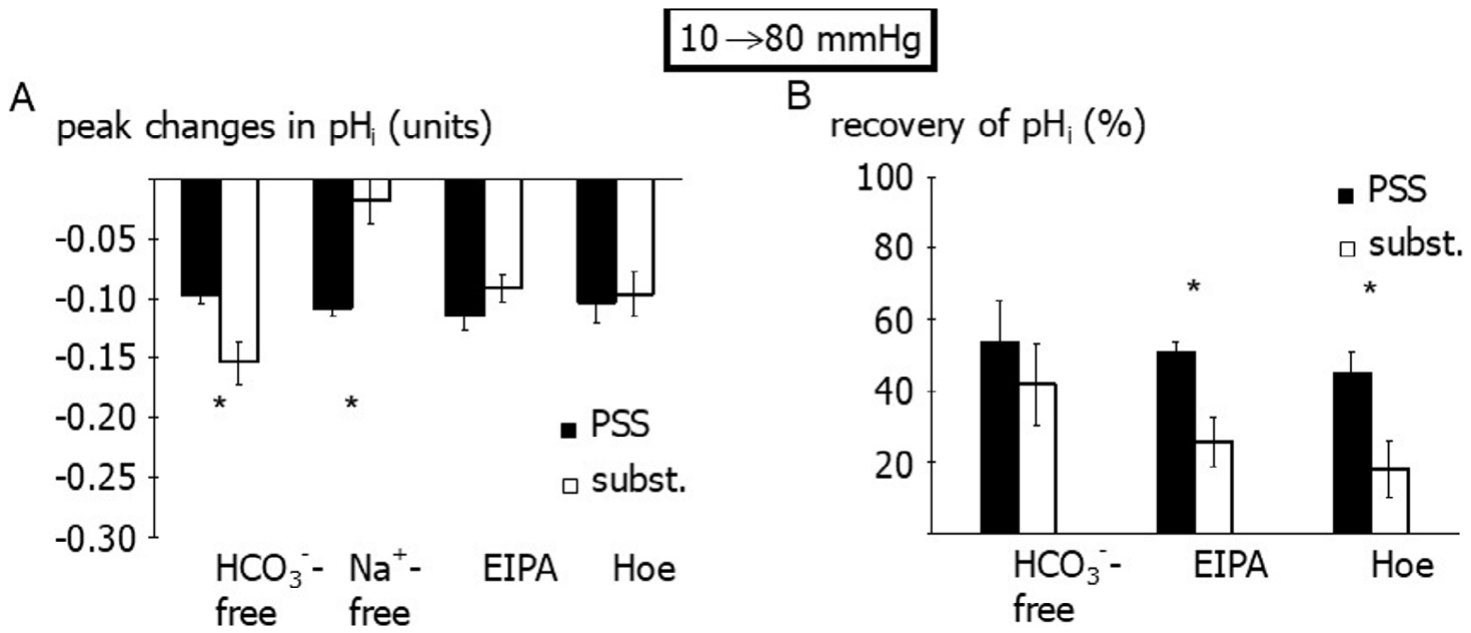
Effect of changes in transmural pressure on pH_i_ during inhibition of sarcolemmal pH_i_ regulating mechanisms. Sarcolemmal pH_i_ regulating mechanisms were inhibited by removal of Na^+^ (Na^+^‐free) or ${\mathrm{HCO}}_{3}^{-}$ (${\mathrm{HCO}}_{3}^{-}$‐free) or with 3 × 10^−5^ M EIPA or 3 × 10^−5^ M Hoe 694. (A) Peak changes of pH_i_ in response to a pressure step from 10 to 80 mmHg. ${\mathrm{HCO}}_{3}^{-}$‐free: *n* = 8, *p < 0.05, Welch's test; Na^+^‐free: *n* = 10, *p < 0.05, Welch's test; EIPA: *n* = 7, *p* = 0.19, Welch's test; HOE: *n* = 8, *p* = 0.74, Welch's test. (B) Recovery of pH_i_ in response to a pressure step from 10 to 80 mmHg. ${\mathrm{CO}}_{3}^{-}$‐free: *n* = 8, *p* = 0.47, Welch's test; EIPA: *n* = 7, *p < 0.05, Mann–Whitney test; HOE: *n* = 8, *p < 0.05, Mann–Whitney test.

Since many of the sarcolemmal pH_i_ regulating mechanisms are coupled to the transport of ${\mathrm{HCO}}_{3}^{-}$ or Na^+^, a first series of experiments was performed using bicarbonate‐ or sodium‐free solutions. In bicarbonate‐free solution, pressure‐induced peak pH_i_ changes were enhanced compared to control in PSS (Figure 3A). However, we did not detect a change in the recovery of pH_i_ (Figure 3B). In Na^+^‐free solution, pressure‐induced peak pH_i_ changes were reduced considerably compared to control in PSS (Figure 3A). Under these conditions, recovery in Na^+^‐free solution could not be assessed.

To further study the mechanisms contributing to the pressure‐induced changes of pH_i_, specific inhibitors of the Na^+^/H^+^ exchanger (NHE) were employed. We did not detect a change in pressure‐induced peak pH_i_ changes in the presence of 30 μM EIPA or 30 μM Hoe694 compared to control in PSS (Figure 3A). However, the recovery of pH_i_ was reduced compared to control in PSS (Figure 3B).

### 2.3 Effect of Inhibition of Sarcolemmal pH_i_ Regulating Mechanisms on Proton Fluxes After an Acid Load

**FIGURE 4:**
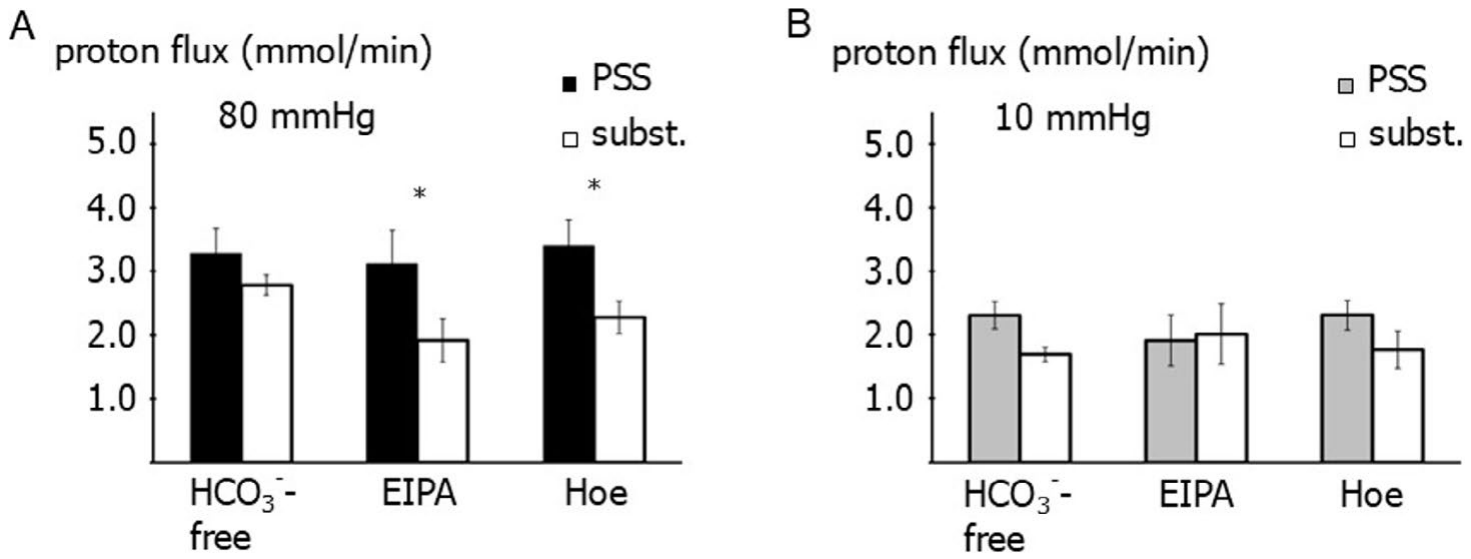
Proton fluxes in response to an acid load during inhibition of sarcolemmal pH_i_ regulating mechanisms. Sarcolemmal pH_i_ regulating mechanisms were inhibited by removal of ${\mathrm{HCO}}_{3}^{-}$ (${\mathrm{HCO}}_{3}^{-}$‐free) or with 3 × 10^−5^ M EIPA or 3 × 10^−5^ M Hoe 694. Proton fluxes were calculated from pH_i_ recovery rates after an acid load and the respective buffer power. The acid load was induced by application of 10 mM NH_4_Cl for 10 min. (A) Proton fluxes at a transmural pressure of 80 mmHg. ${\mathrm{HCO}}_{3}^{-}$‐free: *n* = 9, *p* = 0.44, Welch's test; EIPA: *n* = 7, *p < 0.05, Mann–Whitney test; HOE: *n* = 10, *p < 0.05, Welch's test. (B) Proton fluxes at a transmural pressure of 10 mmHg. ${\mathrm{HCO}}_{3}^{-}$‐free: *n* = 9, *p* = 0.08, Mann–Whitney test; EIPA: *n* = 7, *p* = 0.80, Welch's test; HOE: *n* = 7, *p* = 0.11, Welch's test.

The reduction of the pH_i_‐recovery after a pressure increase from 10 to 80 mmHg by inhibitors of the Na^+^/H^+^ exchanger indicates that the exchanger is able to respond to the acidification induced by pressure elevation. In order to establish whether the capability of the exchanger to respond to an acidification depends on the pressure level, proton fluxes in response to an acid load were determined at 10 and 80 mmHg. Proton fluxes were induced with 10 mM NH_4_Cl before and during inhibition of the exchanger. The proton flux at 80 mmHg (3.28 ± 0.26 mmol/min, *n* = 26) was higher than the proton flux at 10 mmHg (2.20 ± 0.17 mmol/min, *n* = 26; *p* < 0.05, *Welch's* test). In bicarbonate‐free solution we did not detect an alteration of proton fluxes compared to control in PSS at either 10 or 80 mmHg (Figure 4A,B). Further, at 80 mmHg, 30 μM EIPA and 30 μM Hoe 694 reduced the proton flux after an acid load compared to control in PSS (Figure 4A). In contrast, at 10 mmHg we did not detect an effect of the inhibitors on proton flux (Figure 4B).

### 2.4 Effect of Inhibition of Sarcolemmal pH_i_ Regulating Mechanisms on Pressure‐Induced Changes of the Intracellular Calcium Concentration

**FIGURE 5:**
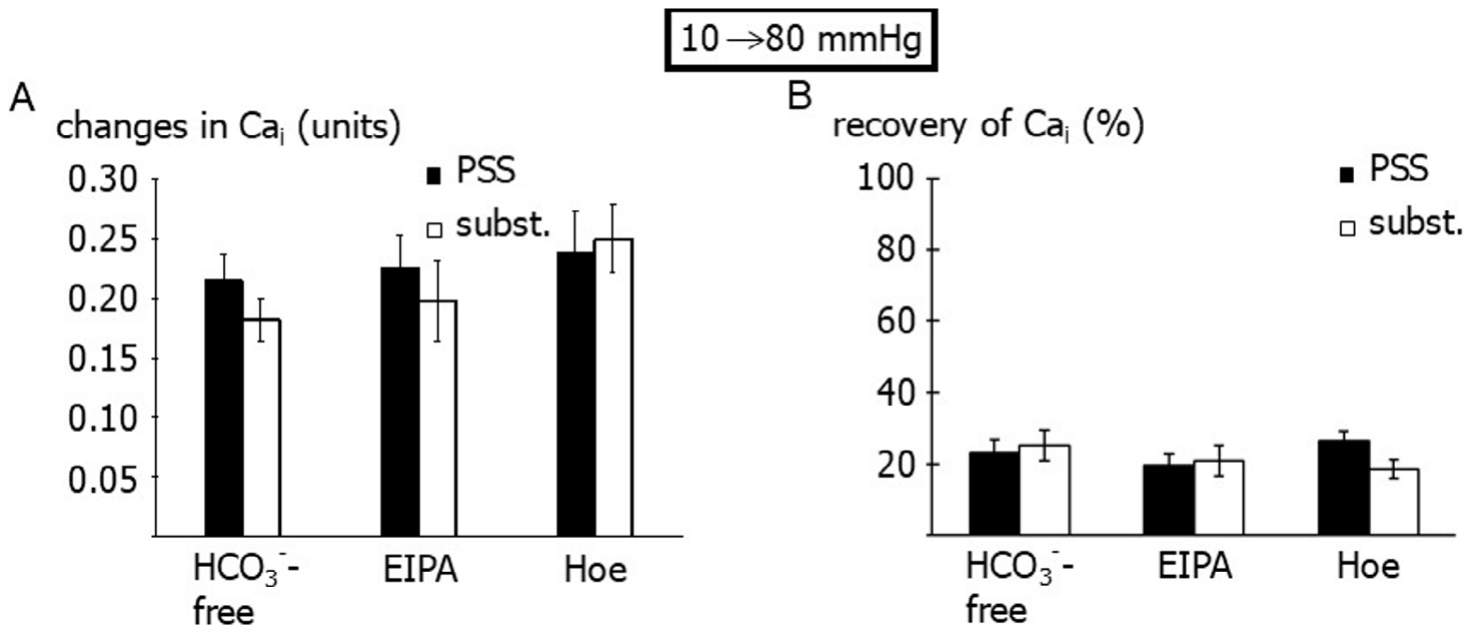
Effect of changes in transmural pressure on Ca_i_ during inhibition of sarcolemmal pH_i_ regulating mechanisms. Sarcolemmal pH_i_ regulating mechanisms were inhibited by removal of ${\mathrm{HCO}}_{3}^{-}$ (${\mathrm{HCO}}_{3}^{-}$‐free) or with 3 × 10^−5^ M EIPA or 3 × 10^−5^ M Hoe 694. (A) Peak changes of Ca_i_ in response to a pressure step from 10 to 80 mmHg. ${\mathrm{HCO}}_{3}^{-}$‐free: *n* = 8, *p* = 0.37, Mann–Whitney test; EIPA: *n* = 5, *p* = 0.55, Welch's test; HOE: *n* = 7, *p* = 0.40, Mann–Whitney test. (B) Recovery of Ca_i_ in response to a pressure step from 10 to 80 mmHg. ${\mathrm{HCO}}_{3}^{-}$‐free: *n* = 8, *p* = 0.65, Mann–Whitney test; EIPA: *n* = 5, *p* = 0.82, Welch's test; HOE: *n* = 7, *p* = 0.07, Welch's test.

In order to understand how pressure‐induced changes in pH_i_ modulate the myogenic response, pressure‐induced changes in intracellular calcium were studied. Under control conditions, the peak Ca_i_ change was 0.23 ± 0.02 ratio units (*n* = 20) upon a pressure increase from 10 to 80 mmHg. Then, Ca_i_ recovered by 24.1% ± 2.1% (*n* = 20). For the pressure increase from 10 to 80 mmHg we did not detect an alteration of pressure‐induced peak Ca_i_ changes or of the recovery of Ca_i_, neither in bicarbonate‐free solution nor by inhibitors of the Na^+^/H^+^ exchanger (Figure 5A,B).

### 2.5 Effect of Pressure‐Induced Changes of pH_i_ on the Myogenic Response

**FIGURE 6:**
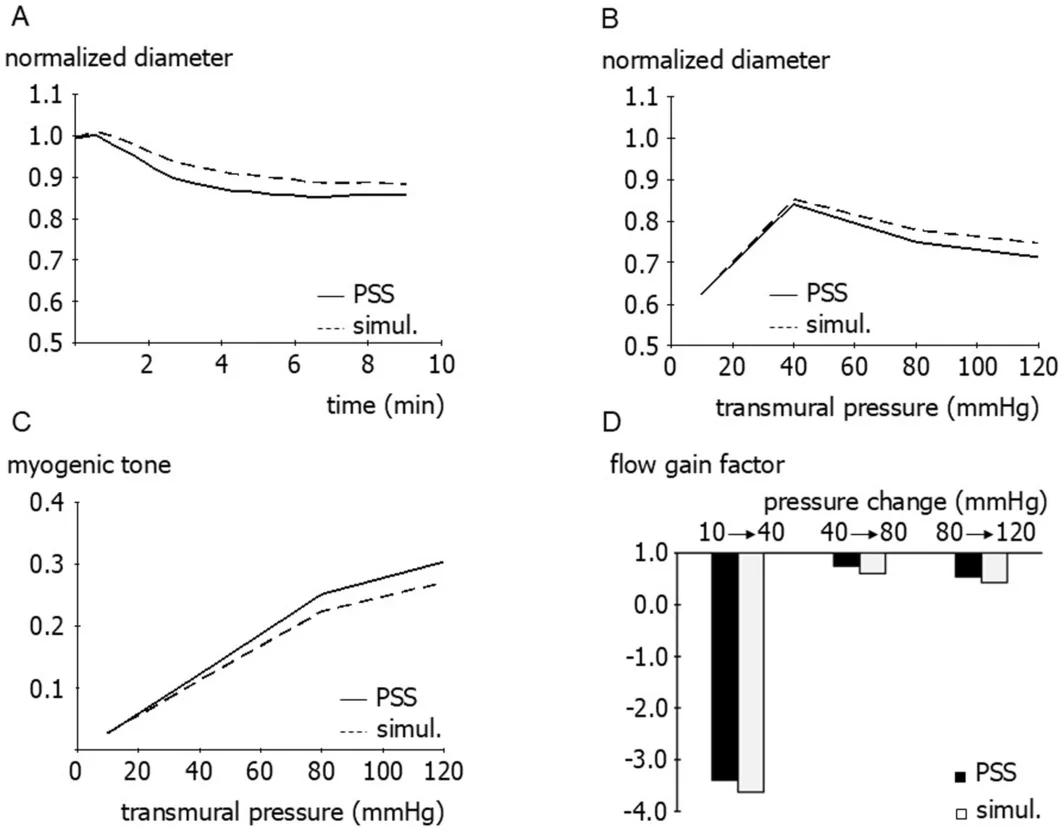
Effect of transmural pressure on vessel diameter and myogenic activity—role of pH_i_. (A) diameter change for a pressure step from 10 to 80 mmHg in PSS and as simulated data supposing no accompanying change(s) in pH_i_. Simulation is calculated from the relation between maximum changes in diameter and pH_i_ for an acid load induced by the washout of 10 mM NH_4_Cl. (B) Effect of transmural pressure on vessel diameter in PSS and as simulated data supposing no accompanying change(s) in pH_i_. (C) Effect of transmural pressure on myogenic tone in PSS and as simulated data supposing no accompanying change(s) in pH_i_. (D) Flow gain factor for different pressure changes in PSS and as simulated data supposing no accompanying change(s) in pH_i_.

The determination of the contribution of pressure‐induced changes of pH_i_ to the myogenic response requires the knowledge of the myogenic response in the absence of associated pH_i_ changes. However, the latter is unknown. Therefore, the myogenic response in the absence of pH_i_ changes was determined semi‐empirically. Briefly, the native myogenic response, that is, pressure‐induced diameter responses and their associated pH_i_ changes, was measured. In addition, the relationship between pH_i_ changes and vessel diameter changes evoked by NH_4_Cl‐induced selective alterations in pH_i_ was obtained experimentally. Based on this relationship, the changes in pH_i_ associated with the native myogenic response were used to calculate pressure‐induced diameter changes in the virtual absence of pH_i_ changes. Analysis of the latter showed that the native myogenic response is faster than the virtual myogenic response in the absence of pH_i_ changes (Figure 6A). The time constant of diameter changes induced by a pressure increase from 10 to 80 mmHg is 114 s for the native and 181 s for the virtual myogenic response. Further, the native myogenic response is stronger than the virtual myogenic response in the absence of pH_i_ changes (Figure 6B) and the corresponding myogenic tone is larger than the virtual myogenic tone in the absence of pH_i_ changes (Figure 6C). In addition, the flow gain factor was between −4 and −3 in the pressure range between 10 and 40 mmHg with weak myogenic constriction and between 0.5 and 1 in the pressure range between 40 and 120 mmHg with strong myogenic constriction (Figure 6D). The flow gain factor characterizes the autoregulation of blood flow during pressure changes [23] based on a Poiseuille model under laminar flow conditions. A flow gain factor of 1 represents perfect flow autoregulation, a flow gain factor > 1 indicates excessive autoregulation, and a flow gain factor < 1 indicates underperforming autoregulation. For all pressure steps tested, the native flow gain factor is closer to 1 than the virtual flow gain factor in the absence of pH_i_ changes (Figure 6D).

## 3 Discussion

The present study shows that the myogenic response is associated with changes in pH_i_. Following a pressure increase, pH_i_ decreased. It initially reached a peak and then recovered partly. The initial pH_i_ peak is augmented in bicarbonate‐free solution, but was not affected by inhibitors of Na^+^/H^+^ exchange. The pH_i_ recovery was not altered in bicarbonate‐free solution, but was reduced by inhibitors of Na^+^/H^+^ exchange. Proton fluxes were larger at 80 mmHg than at 10 mmHg. Bicarbonate‐free solution had no effect on proton fluxes at either 80 mmHg or 10 mmHg. Inhibitors of Na^+^/H^+^ exchange reduced proton fluxes only at 80 mmHg. Pressure‐induced changes in intracellular calcium were not affected by either bicarbonate‐free solution or inhibitors of Na^+^/H^+^ exchange. The data presented suggest that pH_i_ changes induced by a pressure increase facilitate the myogenic response.

### 3.1 Intracellular pH (pH_i_ ) in Vascular Smooth Muscle

The pH_i_ at 10 mmHg in the smooth muscle cells of the small artery used in this study ranged between 7.0 and 7.1. This is in good agreement with previously reported pH_i_ values in the absence of agonists, which ranged from 7.1 to 7.3 regardless of the vessel type studied [18], see also overviews in [12, 15, 21].

### 3.2 Immediate Response of pH_i_ in Vascular Smooth Muscle to an Increase in Transmural Pressure

The present study found that a pressure increase leads to an immediate fall in pH_i_. Thus, myogenic contraction is accompanied by an intracellular acid load. This finding is supported by studies showing intracellular acidification during noradrenaline‐induced contraction in mouse [16] and rat [20] mesenteric arteries, H_2_O_2_‐induced contraction in mouse mesenteric arteries [17], and KCl‐induced contraction in rat mesenteric resistance arteries [18, 19]. Of note, no change in pH_i_ has been reported for vasopressin‐induced [19] and noradrenaline‐induced contraction [18] in rat mesenteric arteries. Thus, our data showing a pressure‐induced fall in pH_i_ reproduce some of the earlier reports demonstrating acidification during vessel contraction and extend the list of factors that can induce such a response beyond vasoactive substances to include a mechanical factor: transmural pressure.

#### 3.2.1 Mechanism of the Pressure‐Induced Immediate Fall in pH_i_

Several mechanisms have been suggested that could be involved in the immediate pH_i_‐response during vasocontraction. In the present study, they were explored by inhibiting sarcolemmal pH_i_ regulating mechanisms.

It is noteworthy that in sodium‐free solution, a strong inhibition of the pressure‐induced immediate fall in pH_i_ was observed. However, the use of sodium‐free solution produced very large changes in vessel diameter and intracellular calcium, which were probably caused by switching the Na^+^/Ca^2+^ exchanger from the forward to the reverse mode [24]. Thus, the pressure‐induced fall in pH_i_ in sodium‐containing and in sodium‐free solution started from considerably different initial conditions. Consequently, the reduction in the pressure‐induced fall in pH_i_ in sodium‐free solution may be due either to the inhibition of sodium‐dependent sarcolemmal pH_i_ regulating mechanisms or to the different initial conditions. The latter explanation seems to be more likely, since the application of bicarbonate‐free solution or of Na^+^/H^+^ exchange‐inhibitors did not reduce the pressure‐induced immediate fall in pH_i_. Nevertheless, the data obtained in sodium‐free solution are very difficult to interpret. Therefore, we did not perform any further experiments in sodium‐free solution.

Regarding the mechanism of the pressure‐induced immediate fall in pH_i_, firstly, there could be competition between protons and calcium ions for binding to internal buffers [19]. This explanation requires a close correlation between pressure‐induced changes in Ca_i_ and pH_i_ given an unchanged buffering power. However, in the present study, we found that bicarbonate removal had no effect on the pressure‐induced increase in intracellular calcium, although it did alter the pressure‐induced fall in pH_i_. Thus, competition between calcium ions and protons for binding sites seems not to explain the pressure‐induced immediate fall in pH_i_.

Secondly, it has been suggested that a fall in pH_i_ could be due to a deactivation of acid extrusion mechanisms. NHE1 is an important Na^+^/H^+^ exchanger in vascular smooth muscle cells, as it contributes to the recovery from an acid load in several arteries [10, 16, 17, 25]. In the present study, inhibitors of Na^+^/H^+^ exchange had no effect on the pressure‐induced immediate fall in pH_i_. Similar results have been reported for KCl‐induced contractions in rat mesenteric arteries [19]. Moreover, we observed that inhibitors of Na^+^/H^+^ exchange reduce proton fluxes evoked in response to a NH_4_Cl‐induced acid load at 80 mmHg but not at 10 mmHg, suggesting a pressure‐induced activation rather than deactivation of NHE1. Thus, deactivation of Na^+^/H^+^ exchange seems not to contribute to the pressure‐induced immediate fall in pH_i_.

Furthermore, NBCn1 is an important Na^+^/${\mathrm{HCO}}_{3}^{-}$ co‐transport in vascular smooth muscle cells, as it contributes to the recovery from an acid load induced experimentally with the NH_4_^+^ prepulse technique in several mouse arteries [9, 16, 26, 27]. In the present study, we observed that in bicarbonate‐free solution, the pressure‐induced immediate fall in pH_i_ was enhanced compared to PSS. Such an enhancement of the pH_i_‐response in bicarbonate‐free solution has previously been observed for KCl‐induced contractions in rat mesenteric arteries [18, 19] and H_2_O_2_‐induced contractions in mouse mesenteric arteries [17]. Additional support for the data of the present study is provided by results showing an appearance of a fall in pH_i_ in bicarbonate‐free solution during noradrenaline‐ and AVP‐induced contractions [18, 19]. The cited data have been interpreted to suggest that Na^+^/${\mathrm{HCO}}_{3}^{-}$ co‐transport prevents the acidification induced by vasoconstrictor substances [18, 28]. However, in the present study, we also observed that bicarbonate removal had no effect on proton fluxes evoked in response to a NH_4_Cl‐induced acid load, neither at 10 mmHg nor at 80 mmHg. This suggests that proton fluxes mediated by Na^+^/${\mathrm{HCO}}_{3}^{-}$ co‐transport play a negligible role at both transmural pressures studied. In conclusion, deactivation of Na^+^/${\mathrm{HCO}}_{3}^{-}$ co‐transport seems not to explain the pressure‐induced immediate fall in pH_i_.

Thirdly, contraction could cause acidification, for example, due to ATP hydrolysis or in association with lactate production [19]. Indeed, a correlation between contraction and acidification was observed in the present study, with a striking similarity in the time course of pressure‐induced changes in diameter and pH_i_. Moreover, no pressure‐induced changes in pH_i_ occurred in vessels that did not show pressure‐induced contractions in calcium‐free solution, supporting a contraction associated acid load. As we did not interfere with crossbridge cycling, alternative sources (e.g., glycolysis, CO_2_ hydration) cannot be fully excluded. Of note, removal of extracellular bicarbonate can weaken the intracellular proton buffer. The buffering power of intracellular proton buffers is quite large in vascular smooth muscle cells, about 30–40 mM/unit pH change at resting values of pH_i_ [12, 29]. This explains the relatively small pressure‐induced changes in pH_i_. Thus, after removal of extracellular bicarbonate, the intracellular buffer may no longer be able to absorb the protons produced during contraction as effectively as in the presence of extracellular bicarbonate. This suggestion is supported by the general finding that disturbances of pH_i_ are limited by pH‐buffering mechanisms in the short time [12]. Moreover, it is consistent with the increased pressure‐induced immediate fall in pH_i_ after bicarbonate removal observed in the present study.

In conclusion, taking all arguments into account, the most likely explanation for the pressure‐induced immediate fall in pH_i_ is a release of protons from the contractile machinery and not an involvement of sarcolemmal pH_i_ regulating mechanisms.

### 3.3 Delayed Response of pH_i_ in Vascular Smooth Muscle to an Increase in Transmural Pressure

The present study found that the immediate fall in pH_i_ associated with a pressure increase was followed by a partial recovery of pH_i_. Thus, the intracellular acid load induced by myogenic contraction is accompanied by regulatory mechanisms that move pH_i_ back toward its initial value. In contrast, KCl‐induced intracellular acidification in rat mesenteric arteries [18, 19] and H_2_O_2_‐induced acidification in mouse mesenteric arteries [17] did not possess a recovery phase. Currently, detailed data on the time course of the effect of vasoconstrictors on pH_i_ are lacking to explain these differences.

#### 3.3.1 Mechanism Contributing to the Recovery After the Pressure‐Induced Immediate Fall in pH_i_

NBCn1 is an important Na^+^/${\mathrm{HCO}}_{3}^{-}$ co‐transport in vascular smooth muscle cells. In the present study, we observed that in bicarbonate‐free solution, recovery from the pressure‐induced immediate fall in pH_i_ was similar to that in PSS. This finding is supported by the observation that Na^+^/${\mathrm{HCO}}_{3}^{-}$ co‐transport‐dependent recovery from an acid load after washout of ${\mathrm{NH}}_{4}^{+}$ was not affected by H_2_O_2_ [17]. Further, we observed that bicarbonate removal had no effect on proton fluxes evoked in response to an NH_4_Cl‐induced acid load at either 10 mmHg or 80 mmHg. In contrast, it has been reported that recovery from an acid load after washout of ${\mathrm{NH}}_{4}^{+}$ was slower in the absence of bicarbonate than in its presence in rat and mouse mesenteric arteries [9, 18] as well as mouse mesenteric, coronary and cerebral arteries [16]. It has been shown that NBCn1 in particular contributes to the recovery from an acid load after washout of ${\mathrm{NH}}_{4}^{+}$ [9, 16, 26, 27], and it has been suggested that the acid load accompanying noradrenaline‐induced constriction was extruded by a calcium‐induced, calcineurin‐dependent activation of NBCn1 [20]. Thus, these data collectively suggest that Na^+^/${\mathrm{HCO}}_{3}^{-}$ co‐transport contributes to the recovery from an acid load induced by washout of ${\mathrm{NH}}_{4}^{+}$ in several arteries, but not in rat small tail arteries. In addition, involvement of Na^+^/${\mathrm{HCO}}_{3}^{-}$ co‐transport in the pH_i_ recovery after a pressure‐induced acidification seems unlikely in this artery.

The Na^+^/H^+^ exchanger NHE1 is another pH_i_‐regulating mechanism in vascular smooth muscle cells. In the present study, we observed that the recovery from the pressure‐induced immediate fall in pH_i_ was considerably reduced by inhibitors of Na^+^/H^+^ exchange. Two different inhibitors showed essentially the same effect, consistent with a specific action of these inhibitors. The concentrations of the inhibitors employed in this study are similar to those used in most studies on the role of the Na^+^/H^+^ exchanger in vascular smooth muscle. Further, we observed that proton flux evoked in response to a NH_4_Cl‐induced acid load was lower at 10 mmHg than at 80 mmHg and that inhibitors of Na^+^/H^+^ exchange reduced proton flux at 80 mmHg but not at 10 mmHg, suggesting a pressure‐induced activation of Na^+^/H^+^ exchange. Our results are supported by observations showing that recovery from an acid load after washout of ${\mathrm{NH}}_{4}^{+}$ was reduced during inhibition of Na^+^/H^+^ exchange in rat and mouse mesenteric arteries [16, 18], that recovery from an acid load after washout of ${\mathrm{NH}}_{4}^{+}$ remaining in bicarbonate‐free solution was abolished in mesenteric arteries of NHE1 knock‐out mice [10] and that NHE1 in particular contributes to recovery from an acid load in several arteries [10, 16, 17, 25]. The pressure‐induced activation of NHE1 is likely caused primarily by the well‐known strong stimulatory effect of intracellular protons on NHE1 [30]. In addition, this activation could be partly attributable to calcium‐ and calcium‐sensitivity‐related signaling pathways involved in the myogenic response, since NHE1 is activated, for example, by calcium/camodulin [31] and Rho‐kinase [32]. Clarification of these hypotheses was beyond the scope of the present study and should be addressed in future studies. In contrast to our finding of pressure‐induced activation of Na^+^/H^+^ exchange, it has been reported that insulin and H_2_O_2_ reduce NHE‐dependent recovery from an acid load after washout of ${\mathrm{NH}}_{4}^{+}$ [17]. Due to a lack of further data on the role of Na^+^/H^+^ exchange in pH_i_ recovery after vasoconstrictor‐induced acidification in intact arteries, the reason for this difference remains to be clarified.

In conclusion, the most likely explanation for the pH_i_ recovery after pressure‐induced acidification is activation of Na^+^/H^+^ exchange without contribution of Na^+^/${\mathrm{HCO}}_{3}^{-}$ co‐transport.

### 3.4 Effect of Pressure‐Induced Changes of pH_i_ on the Myogenic Response

In order to determine how pressure‐induced changes in pH_i_ contribute to the myogenic response, the myogenic response in the absence of associated pH_i_ changes should be identified. Unfortunately, the latter is not known. In the present study, it was not possible to selectively abolish pressure‐induced changes in pH_i_. Therefore, a direct observation of the myogenic response in the absence of pressure‐induced changes of pH_i_ was not achievable. This is a clear limitation of our study. Instead, a semi‐empirical approach was used rather than direct pH_i_ clamp in intact, pressurized arteries. A virtual myogenic response in the absence of changes of pH_i_ was estimated based on the relationship between vessel diameter and pH_i_, which was obtained experimentally after induction of a selective change in pH_i_ using NH_4_Cl. This approach showed that the myogenic response in the presence of changes of pH_i_, that is, the native myogenic response, is faster and stronger than the virtual myogenic response in the absence of changes of pH_i_. Furthermore, the native flow gain factor, which characterizes the autoregulation of blood flow during pressure changes [23] based on a Poiseuille model under laminar flow conditions, is closer to 1 than the virtual flow gain factor in the absence of pH_i_ changes, indicating that pH_i_ improves an index of autoregulatory behavior under our modeling assumptions. A flow gain factor of 1 represents perfect flow autoregulation, a flow gain factor > 1 indicates excessive autoregulation, and a flow gain factor < 1 indicates underperforming autoregulation. Thus, the myogenic response observed in the present study reflected an underperforming autoregulation, although this autoregulation was better for the native compared to the virtual myogenic response.

The conclusion that the myogenic response is stronger when changes of pH_i_ occur is consistent with the observation that the immediate response to a selective fall in pH_i_ in resistance arteries in most preparations is contraction (for overview see reviews [15]) including rat small tail arteries explored in the present study [33]. The contraction observed in response to a selective fall in pH_i_ was often evoked by washout of ${\mathrm{NH}}_{4}^{+}$, which led to a relatively strong intracellular acidification. It has been suggested that the contraction associated with this selective, relatively large fall in pH_i_ is elicited by an increase in intracellular calcium in resistance arteries [34, 35]. In contrast, in the present study, we observed that interventions that affect sarcolemmal pH_i_ regulating mechanisms and alter the pH_i_‐reaction during the myogenic response had no effect on the reaction of the intracellular calcium concentration during the myogenic response. Thus, the effect of the fall in pH_i_ during the myogenic response does not appear to be mediated by an increase in intracellular calcium. Interestingly, a study on permeabilized rat main tail arteries showed an acidosis‐induced increase in calcium sensitivity, presumably due to a decreased phosphatase activity [36]. Furthermore, acute intracellular acidosis evoked under non‐physiological conditions (inhibition of Na^+^/H^+^ exchange in the absence of extracellular bicarbonate) strengthened the myogenic response without affecting the pressure‐induced change in intracellular calcium in rat coronary arteries [37]. Of note, a weakening of the myogenic response was observed during chronic, long‐term intracellular acidosis caused by NBCn1 deletion in mouse middle cerebral arteries. This effect was suggested to be mediated by reduced calcium sensitivity due to an inhibition of Rho‐kinase at lower pH_i_ [9, 10, 16, 27]. Interestingly, there may be a unifying explanation for the contradiction between the effects of acute and chronic acidosis. It has been shown that the myogenic response of the small tail artery, the vessel used in the present study, is associated with Rho‐kinase‐dependent changes in calcium sensitivity [38]. Rho‐kinase activity has been reported to possess a bell‐shaped dependence on pH_i_ [9]. Importantly, the chronic decrease in pH_i_ resulting from NBCn1 deletion was approximately 0.3 units, whereas during acute pressure changes, as reported in the present study, it was no more than 0.1 units. These data are consistent with the idea that the small fall in pH_i_ during acute pressure changes climbs up the bell‐shaped curve of Rho‐kinase activity, leading to the suggested increase in calcium sensitivity. The larger fall in pH_i_ during long‐term changes in pH_i_ climbs over the bell‐shaped peak of the curve of Rho‐kinase activity and then down to lower levels of activity, leading to the reported decrease in calcium sensitivity. Clarification of this hypothesis was beyond the scope of the present study and should be addressed in future studies.

## 4 Conclusion

In conclusion, this study showed that in rat small tail arteries a pressure increase is accompanied by a decrease in pH_i_ that appears to facilitate the myogenic response. The results of this study suggest that the pressure‐induced immediate fall in pH_i_ is caused by a release of protons from the contractile machinery, and that the subsequent pH_i_ recovery is due to activation of Na^+^/H^+^ exchange.

## 5 Material & Methods

The methods used in this study will be described briefly; they have been presented in detail previously [39, 40].

### 5.1 Dissection and Mounting of the Vessels

The investigation conforms with the European Convention on the protection of animals used for scientific purposes and ARRIVE guidelines 2.0. Approval for the use of laboratory animals in this study was granted by a governmental committee on animal welfare (LALLFM‐V/PSD/7221.3‐2.3‐028/06).

All experiments were performed on isobaric preparations of small tail arteries (first order branches of the main ventral tail artery see also [41]) of male Wistar Kyoto rats under non‐flow conditions. To ensure better comparability with our previous results [38, 41], the experiments in the present study were conducted exclusively on male rats. This represents a limitation of our study. This vessel was selected because of its exceptionally strong myogenic response. Vessels were mounted in an experimental chamber containing experimental solution (PSS, composition see below). Leaking vessels were discarded at any stage of the experiment to ensure non‐flow conditions. The microscope image of the vessel was viewed with a CCD camera and digitized with a frame grabber board [39]. Vessels were transilluminated with red light at low intensity in order to perform both videomicroscopic diameter determination and fluorescence measurement of intracellular pH or calcium concentration simultaneously (see below).

### 5.2 Experimental Protocol

After mounting of the vessel, it was pressurized to 80 mmHg, the temperature was set to 37.0°C ± 0.5°C and the pH to 7.40 ± 0.05. When a spontaneous myogenic tone had developed, vessel viability was tested with 1 μM acetylcholine to test endothelial cell function and 0.1 μM norepinephrine to test smooth muscle cell function. Only vessels showing a complete dilation in response to acetylcholine and an at least 20% constriction in response to norepinephrine were used in the experiments. The ability of the vessel to generate myogenic responses was tested by pressure steps from 10 to 80 mmHg. At the end of the experiment, the diameter of the relaxed vessel at 80 mmHg was determined with relaxation solution. All drugs (see below) were applied to the vessels' adventitial side only.

### 5.3 Determination of Intracellular pH

Fluorescence measurements were performed at excitation wave lengths of 455 and 493 nm and the emitted light was filtered at 505 nm and detected by a photomultiplier. After the viability tests and determination of the background fluorescence of the unloaded vessel and the equipment (later subtracted from the corresponding fluorescence signals), vessels were loaded with 1 μM BCECF‐AM at 37°C for 20 min followed by 20 min washout in complete darkness. Measurements of pH_i_ were always completed with a calibration of the fluorescence signal in terms of pH [18, 20, 42, 43]. Briefly, the vessel was incubated with 2 mg/L nigericin in calibration solution. Then, the chamber was successively perfused with calibration solution at pH of 7.0, 7.4, and 7.8. Steady state values of emission were used for calculation of a calibration curve. Based on these data, the emission ratio was transformed into values of pH_i_. Changes in pH_i_ were subjected to standard analysis procedures. Briefly, the pH_i_‐recovery rate was determined as the slope of the pH_i_ versus time curve that follows the peak pH_i_ change during washout of 10 mM NH_4_Cl. Proton fluxes were calculated as the product of the pH_i_‐recovery rate and the buffering power of the smooth muscle cells. Buffering power was calculated using the ΔpH_i_ immediately after removal of 10 mM NH_4_Cl (pH regulation was corrected for by back‐extrapolating the pH_i_ measurement) and ΔH^+^ calculated employing the Henderson–Hasselbalch equation [9, 29, 44, 45]. Buffering power was in the range between 27 and 40 mM; we did not detect differences in buffering power between 10 and 80 mmHg or between control and intervention (in particular: repeated measures two‐way ANOVA; bicarbonate‐free: *n* = 9, 10 mmHg vs. 80 mmHg *p* = 0.93, control vs. bicarbonate‐free *p* = 0.66; EIPA: *n* = 7, 10 mmHg vs. 80 mmHg *p* = 0.39, control vs. EIPA *p* = 0.58; HOE: *n* = 10, 10 mmHg vs. 80 mmHg *p* = 0.22, control vs. HOE *p* = 0.51).

### 5.4 Determination of Intracellular Calcium Concentration

Fluorescence measurements were performed at excitation wavelengths of 340 and 380 nm, and the emitted light was filtered at 520 nm and detected by a photomultiplier. After the viability tests and determination of the background fluorescence of the unloaded vessel and the equipment, vessels were loaded with 5 μM FURA‐2‐AM at 37°C for 60 min followed by 20 min washout in complete darkness. Intracellular calcium was estimated by calculating the ratio of the emission signals excited at 340 and 380 nm (F_340_/F_380_) after background subtraction. A calibration of the fluorescence ratio in terms of calcium was not performed in view of the many uncertainties related to the binding properties of FURA‐2 with calcium inside cells of intact vessel preparations [40].

### 5.5 Solutions and Substances

The following solutions were used:

1. PSS (physiological saline solution, experimental solution) in mM: NaCl 120, NaHCO_3_ 26, KCl 4.5, NaH_2_PO_4_ 1.2, MgSO_4_ 1.0, CaCl_2_ 1.6, EDTA 0.025, glucose 5.5, HEPES 5.0, equilibrated with carbogen (95% O_2_ and 5% CO_2_).
2. Solution for vessel preparation: NaCl 145, KCl 4.5, NaH_2_PO_4_ 1.2, MgSO_4_ 1.0, CaCl_2_ 0.1, EDTA 0.025, HEPES 5.0.
3. Relaxation solution (calcium free solution): NaCl 120, NaHCO_3_ 26, KCl 4.5, NaH_2_PO_4_ 1.2, MgSO_4_ 1.0, EGTA 1.0, glucose 5.5, HEPES 5.0, equilibrated with carbogen (95% O_2_ and 5% CO_2_).
4. pH_i_ calibration solution: NaCl 10.0, KCl 144.5, MgCl_2_ 1.0, EGTA 1.0, glucose 5.5, HEPES 10.0.
5. ${\mathrm{HCO}}_{3}^{-}$‐free solution: PSS with equimolar replacement of NaHCO_3_ with NaCl.
6. Na^+^‐free solution: PSS with equimolar replacement of NaCl with choline chloride and of NaHCO_3_ with choline bicarbonate.

For all solutions, pH was set to 7.40 ± 0.05 at 37°C except for preparation solution (7.30 ± 0.05 at 4°C).

All substances for the solutions as well as norepinephrine, acetylcholine, ammonium chloride (NH_4_Cl), choline salts, nigericin, and EIPA (5‐(N‐Ethyl‐N‐isopropyl)amiloride) were obtained from Sigma (Deisenhofen, Germany). Hoe 694 (3‐methylsulfonyl‐4‐piperidinobenzoyl‐guanidine methansulfonate) was provided by Aventis Pharma (Frankfurt/M., Germany). BCECF‐AM (2′,7′‐bis‐(2‐carboxyethyl)‐5‐(and‐6)‐carboxyfluorescein, acetoxymethyl ester) and FURA‐2‐AM were purchased from Molecular Probes (Leiden, The Netherlands).

### 5.6 Statistics

All data are means ± SEM; *n* is the number of vessels. Only one vessel was taken from each rat, thus *n* represents the number of biological replicates. Statistical analysis was performed using GraphPad Prizm 10.1.2. The normality of the data distribution was tested using the Shapiro–Wilk test. Depending on the results of the normality test, either parametric or nonparametric tests were used; the specific tests are indicated in the corresponding figure legends.

## Author Contributions

**Ulrike Krien:** conceptualization, investigation, writing – review and editing, visualization, methodology, formal analysis, writing – original draft, resources. **Rudolf Schubert:** conceptualization, methodology, formal analysis, supervision, resources, project administration, visualization, funding acquisition, writing – original draft, writing – review and editing.

## Funding

The authors have nothing to report.

## Conflicts of Interest

The authors declare no conflicts of interest.

## Data Availability

The data that support the findings of this study are available from the corresponding author upon reasonable request.
